# Determination of Sustainable Critical Flux through a Long-Term Membrane Resistance Model

**DOI:** 10.3390/polym15102319

**Published:** 2023-05-16

**Authors:** Rongle Xu, Yaobo Fan, Min Yang, Jinqiu Song

**Affiliations:** 1 Scientific Research Academy of Guangxi Environmental Protection, Nanning 530022, China; 2 School of Resources, Environment and Materials, Guangxi University, Nanning 530004, China; 17745056451@163.com; 3 Research Center for Eco-Environmental Sciences, Chinese Academy of Sciences, Beijing 100085, China; ybfan@rcees.ac.cn (Y.F.); yangmsunb@126.com (M.Y.)

**Keywords:** sustainable critical flux, membrane resistance model, polymer film fouling

## Abstract

A long-term membrane resistance model (LMR) was established to determine the sustainable critical flux, which developed and simulated polymer film fouling successfully in a lab-scale membrane bioreactor (MBR) in this study. The total polymer film fouling resistance in the model was decomposed into the individual components of pore fouling resistance, sludge cake accumulation and cake layer compression resistance. The model effectively simulated the fouling phenomenon in the MBR at different fluxes. Considering the influence of temperature, the model was calibrated by temperature coefficient τ, and a good result was achieved to simulate the polymer film fouling at 25 and 15 °C. The relationship between flux and operation time was simulated and discussed through the model. The results indicated that there was an exponential correlation between flux and operation time, and the exponential curve could be divided into two parts. By fitting the two parts to two straight lines, respectively, the intersection of the two straight lines was regarded as the sustainable critical flux value. The sustainable critical flux obtained in this study was just 67% of the critical flux. The model in this study was proven to be in good agreement with the measurements under different fluxes and different temperatures. In addition, the sustainable critical flux was first proposed and calculated in this study, and it was shown that the model could be used to predict the sustainable operation time and sustainable critical flux, which provide more practical information for designing MBRs. This study is applicable to polymer films used in a wide variety of applications, and it is helpful for maintaining the long-term stable operation of polymer film modules and improving the efficiency of polymer film modules.

## 1. Introduction

Membrane bioreactors (MBRs) offer a number of advantages over the conventional activated sludge process, such as excellent quality effluent, a more compact treatment facility, a more concentrated biomass and a reduced sludge yield [1]. In the application of wastewater treatment and water reclamation, MBRs have progressively gained acceptance and popularity in China [2]. However, polymer film fouling remains a big challenge for widespread applications of MBRs, which can reduce polymer film flux at a given transmembrane pressure (TMP), or conversely, increase the TMP at a given flux, and finally, increase the energy consumption and costs of the wastewater treatment and water reclamation.

Membrane cleaning is important to reduce membrane contamination and improve membrane performance. Gul et al. [3] reviewed the fouling and cleaning process in microfiltration membranes. Polymer film fouling in a submerged MBR can be attributed to both polymer film pore clogging and sludge cake deposition on the polymer film surface [4]. Numerous studies have directly elucidated the fouling behaviors, and the effects of the key operation conditions and sludge characteristics on polymer film fouling. Recent experimental results indicate that polymer film resistance is mainly affected by aeration turbulence and sludge characterization [5,6,7,8]. Mixed liquor suspended solids (MLSS) and colloids are the main contributors to polymer film resistance [9]. Polymer film pore clogging could be caused by the adsorption of dissolved or colloidal matters, and soluble microbial products (SMP) have been proved to be the dominant pollutant causing pore blocking during long-term operation [10,11]. MLSS were found to be the main contributor to the cake layer [12]. Aeration has a positive effect on cake layer removal, and a higher air flow rate could promote the back transport of deposited materials from the polymer film surface by turbulent shear [13] but with higher energy consumption.

Operation flux is an important parameter in MBR designing and operation, and it is a direct factor in polymer film fouling. Hung et al. [14] reviewed recent studies on membrane compaction, wetting and fouling, demonstrating that an ultra-low-pressure membrane (ULPM) is effective for long-term filtration at stable fluxes. It has been suggested that submerged MBRs should be operated at a flux below the “critical flux”, the so-called subcritical flux, to maintain a sustainable permeability and to mitigate polymer film pollution [15,16,17]. The critical flux or subcritical flux is a variable with a relatively wide range, and there is no clear criterion for accurate subcritical flux determination. For an MBR system, even operating at subcritical fluxes there are significant differences in the polymer film fouling rates and the community structures of the sludge layer that adhere to the polymer film surface [18,19,20]. What is more, based on the sustainable critical flux (flux_sc_), the necessary polymer film area can be determined. In the MBR, too much polymer film, which is determined by a lower flux, increases the investment, but not enough polymer film, which is determined by a higher flux, reduces the output at the designed scale and leads to difficulty maintaining a stable performance on the high flux compared with the flux_sc_. To maintain an MBR’s long-term stable operation, it is important to predict the behaviors of polymer film fouling and cake formation during its long-term operation, and it is necessary to limit the scope of subcritical fluxes, and thus find a sustainable critical flux.

A polymer film filtration model is able to accurately express the polymer film fouling characterization, which allows for determination of the independent fouling characteristics and a better fundamental understanding of the controlling mechanisms. Model-based analysis is an essential tool for polymer film fouling control and the long-term stable operation of an MBR. The polymer film filtration or polymer film fouling models can be classified into two groups, the mechanistic models and the mathematic models. The mechanistic models are based on physical filtration laws, and resistances-in-series is the most common approach [21,22]. The mathematic models based on statistical analysis are used to reveal the relationship among aeration, flux and sludge characterization [23,24]. However, some of the models are restricted in relatively short-term periods, and others cannot quantify the contributions of polymer film foulants to overall polymer film fouling. Moreover, most of these models can neither predict the trend of long-term polymer film fouling nor guide the design and the operation of an actual MBR.

The objective of this study is to predict the sustainable critical flux of A^2^/O-MBR by establishing a long-term membrane resistance model. The sustainable critical flux could be used as operating parameter, which could extend membrane service life and control membrane fouling effectively.

## 2. Theories and Models

In this study, the resistance R was calculated according to Darcy’s law,

(1) R=TMP/μJ

where R is the resistance, m^−1^, and TMP is the pressure, kPa. μ is the viscosity of the sludge suspension, mPa•s. J is the flux, m^3^/(m^2^•d).

The long-term membrane resistance model established in this study is based on a two-stages hypothesis. At the first stage, the specific flux declined rapidly and the operation TMP, $TMP_{a}$, grew slowly along with the operation time; the polymer film pore clogging caused by SMP and the cake layer formation were the main polymer film fouling processes. At the second stage, as $TMP_{a}$ reached a certain value (the critical TMP, $TMP_{c}$), the specific flux declined slowly and $TMP_{a}$ rose rapidly; cake layer compression was the main polymer film fouling process.

### 2.1. Components of Filtration Resistance

The total resistance R comprises three resistance components, including the intrinsic resistance of the polymer film, $R_{m}$, the pore fouling resistance caused by solute deposition inside the polymer film pores, $R_{p}$ and the resistance of the sludge cake layer, $R_{c}$, which is formed by the resistance of sludge cake accumulation $R_{ac}$ and the resistance of cake layer compression $R_{comp}$. R, $R_{m}$, $R_{p}$, $R_{ac}$ and $R_{comp}$, m^−1^.

When $TMP_{a}<TMP_{c}$,

(2) $$R=R_{m}+R_{p}+R_{ac}$$

and when $\;TMP_{a}\;\geq \;TMP_{c}$,

(3) $$R=R_{m}+R_{p}+R_{comp}$$

### 2.2. Polymer Film Pore Fouling Resistance ($R_{p}$)

The pore fouling resistance is expected to increase proportionally to the volume of water production during filtration [25]; that is,

(4) $$R_{p}=r_{p}JCt$$

where $r_{p}$ is the specific pore fouling resistance, m/kg. C is the concentration of polymer film pore foulants, kg/m^3^. J  is the operation flux, m^3^/(m^2^•h). t is the operation time, h.

Many studies have shown that SMP is the main foulant of pore clogging [11,26]; thus, in this paper, we chose the SMP to calculate the $R_{p}$.

(5) $$R_{p}=r_{SMP}JC_{SMP}t\theta$$

where $r_{SMP}$ is the specific pore fouling resistance, m/kg. $C_{SMP}$ is the concentration of polymer film pore foulants, kg/m^3^. θ is the coefficient of polymer film pore clogging caused by SMP.

In this study, through the filtration experiments with different sludge components, SMP was also proved to be the main factor contributing to polymer film clogging, of which the contribution rate could be more than 50%, and the value of θ was 1~2, which was influenced by the suction drag force.

### 2.3. Sludge Cake Accumulation Resistance ($R_{ac}$)

The resistance of the sludge cake layer, $R_{ac}$, was calculated by Equation (5),

(6) $$R_{ac}=r_{c}\times M_{C}$$

where $r_{c}$ is the specific filtration resistance of the sludge cake layer, m/kg. $M_{C}$ is the amount of MLSS accumulating on the polymer film surface, kg/m^2^. $M_{C}$ is a variable changed along with the operation time, and affected by the aeration. The calculation of $M_{C}$ is discussed with the sludge particle being deposited on the polymer film surface and detaching from the polymer film surface.

1. Probability of suspended solids being deposited on the polymer film surface (*E*).

**Figure 1:**
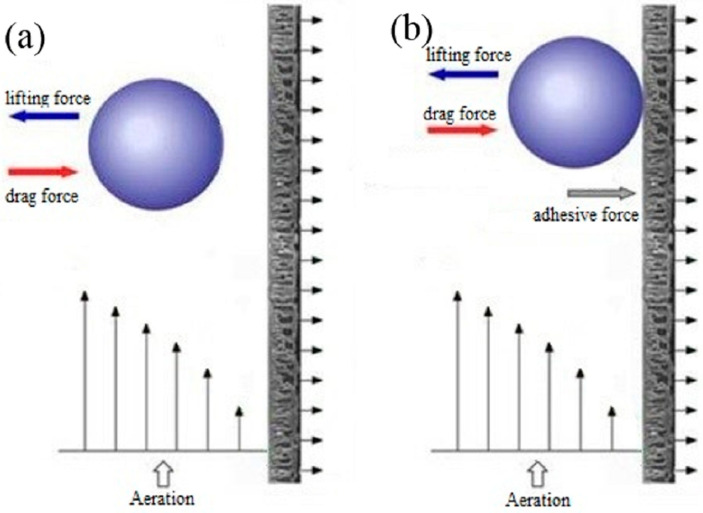
Force diagram of sludge particle. (**a**) Sludge particle being deposited on the polymer film surface, (**b**) sludge particle attached to the polymer film surface.

Two opposite forces regulate the probability (*E*) of the sludge particle being deposited on the polymer film surface, the drag force and the lifting force, of which the drag force caused by suction leads to the attachment, and the lifting force caused by the turbulent flow prevents the sludge particles from attaching to the polymer film surface (Figure 1a). *E* can be estimated [27] as

(7) $$E=\frac{24J}{24J+K_{1}G}$$

where $K_{1}$ is a constant that is related to the lifting force of the sludge particle and particle size, m. $K_{1}$ = 4 × 10^−6^ m [27], and G is the shear intensity on the polymer film surface, s^−1^, which can be determined by Equation (8).

(8) $$G={\left(\frac{\rho gq}{\mu }\right)}^{1/2}$$

g is the gravitational constant, 9.81 m/s^2^. q is the aeration intensity, L/(m^2^•s). ρ is the density of biomass particles, kg/m^3^ and μ is the viscosity, Pa•s.

It is noted that the sludge particles can be affected by the drag force, and the depositing of the sludge particles on the polymer film surface is only easier when the particle size is less than 100 μm [28]. Thus, a coefficient ε, the percent of the sludge practice size less than 100 μm, was introduced into the model.

Above all, the rate of biomass attachment onto the polymer film surface can be written as

(9) $$\frac{dM_{c}}{dt}=ECJ\varepsilon$$

where C is the sludge concentration, kg/m^3^. E is the probability of the suspended solids being deposited on the polymer film surface (Equation (7)), J is the operation flux, m^3^/(m^2^•h), and ε is the percentage of the sludge practice size less than 100 μm.

2. Probability of the suspended solids detaching from the polymer film surface (K).

When a sludge particle attaches on the polymer film surface, there are three forces regulating the probability, the drag force, the lifting force and the adhesive force caused by the stickiness between the biomass particles and the polymer film surface or between the biomass particles (Figure 1b).

However, there is a boundary layer adjacent to the polymer film surface where the velocity is quite low, with a very weak lifting force for the biomass particles to be flushed off from the polymer film surface. Hence, a coefficient γ was introduced into the model to express the ratio of the velocity in the boundary layer (V_bl_) to that of the bulk flow (V_bf_). A stickiness value α was introduced, and 1 − α indicates the actual scouring coefficient after overcoming the adhesion force between the particles and the polymer film surface. Three forces regulate the probability (K) of the sludge particle detaching from the polymer film surface, and K can be estimated by

(10) $$K=\frac{\gamma K_{1}G}{\gamma K_{1}G+24J}\left(1-\alpha \right)$$

where α is the stickiness between the sludge particle and the polymer film surface, α=0.1 [29]. γ is the ratio of V_bl_ to V_bf_.

The rate of biomass detachment from the polymer film surface can be determined by

(11) $$\frac{dM_{f}}{dt}=KCJ\varepsilon$$

where C is the sludge concentration, kg/m^3^, *K* is the probability of SS detaching from the polymer film surface, J is the operation flux, m^3^/(m^2^•h), and ε is the percentage of the sludge practice size less than 100 μm.

The $M_{C}$ in Equation (5) can be determined by Equations (8)–(10) and by integrating $d(M_{c}-M_{f})$, and finally, the sludge cake accumulation resistance. Equation (5) can be established as

(12) $$R_{c}=r_{c}\left(Et_{1}-Kt_{2}\right)CJ\varepsilon$$

where $t_{1}$ is the time of polymer film suction and $t_{2}$ is the aeration time.

### 2.4. Cake Layer Compression Resistance ($R_{comp}$)

The sludge cake is highly compressible and its properties vary with the sharp increase in the applied TMP. Cake layer compression may lead to an increase in the sludge specific resistance, $r_{comp}$, which can be described by the empirical Equation (13) [30],

(13) $$r_{comp}=r_{c}{\left(1+\frac{TMP_{a}}{TMP_{c}}\right)}^{n}$$

where $TMP_{c}$, the critical TMP, is the TMP at which $r_{c}$ is double its initial value, and the value of $TMP_{c}$ varies with the sludge type, sludge conditioning, floc size, etc. [31]. $TMP_{a}$ is the applied TMP. The compressibility coefficient, n, value is in the range of 0 to 1, and for activated sludge, n=1 [31]. At the stage of cake layer compression, the sludge biomass is supposed to cover the polymer film surface fully. Therefore, because of the higher stickiness of sludge biomass, the value of the stickiness coefficient α is set to 0.5 [32].

Based on Equations (12) and (13), the cake layer resistance at the compression stage is expressed as Equation (14),

(14) $$R_{comp}=r_{comp}\left(Et_{1}-Kt_{2}\right)\mathrm{CJ}\varepsilon$$

## 3. Experiments

### 3.1. Lab-Scale Setup

**Figure 2:**
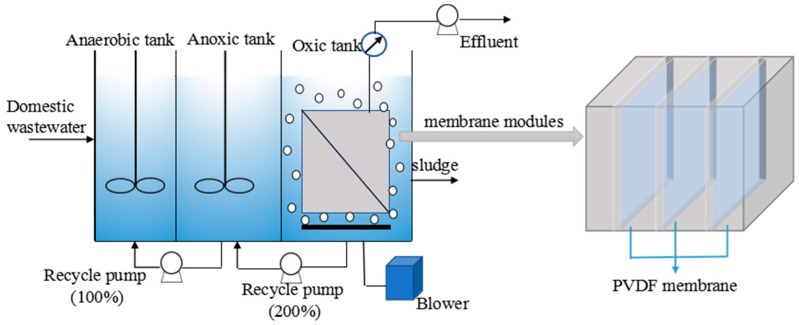
Schematic of lab-scale A^2^/O-MBR reactors.

A submerged A^2^/O-MBR was operated to characterize the polymer film fouling under various conditions (Figure 2). The experimental setup was made up of an anaerobic tank, an anoxic tank and an oxic tank with submerged polymer film modules. The working volume of the A^2^/O-MBR was 58.3 L, including anaerobic tank 8.3 L, anoxic tank 16.7 L and aerobic tank 33.3 L. The anaerobic and anoxic tanks were mixed with a low-speed mixer and air was introduced via perforated pipe under the polymer film module in the oxic tank. Three pieces of flat-sheet PVDF polymer films (0.1 m^2^ per polymer film element, membrane pore size 0.2 µm, SINAP membrane S&T Co., Shanghai, China) were used in the oxic tank of A^2^/O-MBR.

The influent to the MBR was domestic wastewater taken from the sewer at residential district of Research Center for Eco-Environmental Science, Chinese Academy of Science. The sludge residence time (SRT) in A^2^/O-MBR was approximately 20 days or longer. The sludge in the MBR was maintained at a steady state for more than 2 months before experiments, and the fouling experiments lasted more than 2 months. The sludge concentration was about 6 g/L.

Critical flux was determined by the flux-step method [16]. Under trial condition, the critical flux was 30 LMH. Model simulation was carried out with two-stage experiments. The first was operated at different flux under average temperature 15 °C. The filtration flux was set under critical flux, and 10, 18 and 25 LMH were adopted. The other was operated at 10 LMH under average temperature 25 °C. The polymer films operated under a 6 min filtration and 1 min stop cycle; no backwashing of the polymer film was required. The aeration intensity was fixed at 0.83 L/(m^2^•s).

During the test, the increase in the TMP was recorded, along with the running time and water production. The filtration flux of the water production was monitored every day. The MBR stopped when the TMP reached 30 kPa, and then the polymer film module was cleaned thoroughly by physical and chemical methods. Polymer film resistance analyses was calculated by resistance series models in Feng’s study [33]. The irreversible resistance that could not be cleaned by physical and chemical methods was ignored in this study because the new polymer film was used. The physical washing removed cake layer by showering with tap water and sponge swabbing; by this step, the R_c_ was calculated. Chemical washing removed the foulants from polymer film pore, and the R_p_ was calculated by this step.

### 3.2. Model Parameters’ Determination

#### 3.2.1. R_p_ and r_c_

According to ultrafiltration cup experiment, there is a linear relationship between filter time and volume within a certain range. Specific filtration resistance r can be described as [8]

(15) $$\frac{t}{V}=\left(\frac{\mu rC}{2PA^{2}}\right)V+\frac{\mu R_{m}}{PA}$$

The slope b = $\frac{\mu rC}{2PA^{2}}$, r = $\frac{2PA^{2}b}{\mu C}$

where μ is the permeate viscosity, mPa•s. C is the concentration of mixed liquor suspended solids, kg/m^3^. A is the effective filtration area, m^2^. P is the filtration pressure, Pa. t is the filtration time, s. V is the filtration volume, m^3^.

#### 3.2.2. Ratio of Boundary Velocity to Bulk Flow Velocity

A simulation of the 3D flow field between polymer film sheets by the commercial CFD code ANSYS Fluent^®^ showed that there is non-uniformity of gas–liquid flow in channels between two polymer film elements; the flow velocity is higher in the middle of the channel and that much lower in the boundary layer at the polymer film surface [34]. According to the simulation, the ratio of boundary velocity to bulk flow velocity γ is selected at 0.02.

#### 3.2.3. Temperature Coefficient τ

Temperature is a key factor influencing the microbial community, sludge morphology and the filtration of mixed liquid in MBR operation [35]; interactions between temperature, sludge characterization and polymer film fouling are of a complex nature [36]. Low temperature might increase mixed liquor viscosity and reduce particle size, which causes lower particle back transport velocity, and furthermore, leads to negative influence on the polymer film performance [35,37]. Considering the complex role of temperature in polymer film fouling, it is necessary to introduce a coefficient for temperature calibration. Thus, a coefficient τ was introduced in the cake resistance calculation; the Equations (12) and (13) can be changed to

(16) $$R_{c}=r_{c}\left(Et_{1}-Kt_{2}\right)\mathrm{CJ}\varepsilon \tau$$

(17) $$R_{comp}=r_{comp}\left(Et_{1}-Kt_{2}\right)\mathrm{CJ}\varepsilon \tau$$

Some studies suggested differences in permeability of 50% were found between summer and winter periods in the full-scale MBR [37,38]. A lab-scale study also found the differences in permeate flux and permeability between the low temperature and high temperature were from 30% to 80% [39]. Therefore, in this study, the value of τ was set as 0.3.

**Figure 3:**
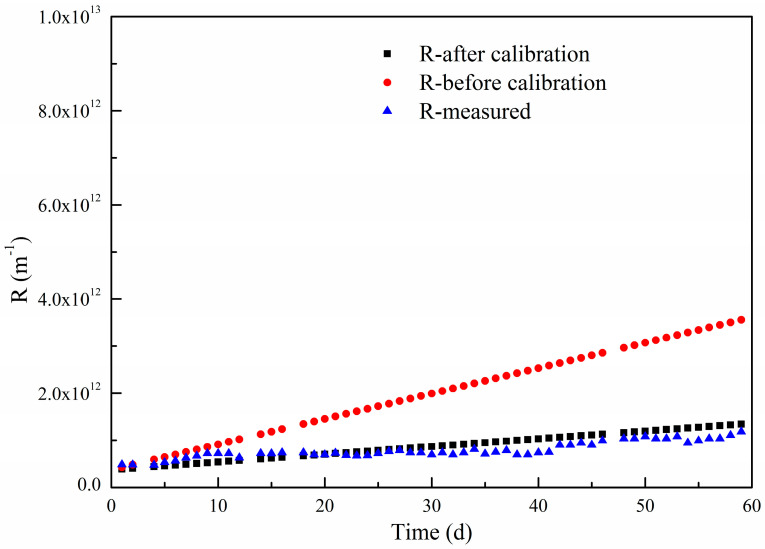
Temperature calibration of long-term membrane resistance model.

In this study, a lab-scale experiment was carried out to prove the effect of temperature with flux set at 10 LMH and temperature at 25 °C. The total polymer film fouling resistances under different simulations are shown in Figure 3 and Table 1. It can be seen that after calibration by coefficient τ, the model simulates the real performance very well, the average deviation between simulated and measured values is 7.18%, which means that the influence of temperature on polymer film fouling can be reflected by coefficient τ.

The parameters and coefficients for model were selected based on laboratory tests, assumptions and literature reports (Table 2). The sludge concentration, SMP concentration and μ were averages of measurements taken during the whole test. SMP was filtered (0.2 μm, cellulose acetate polymer film) and measured TOC (Vario TOC, Elementar Analysensysteme GmbH, Germany). ε, the percent of sludge practice size less than 100 μm, was tested by laser particle size analyzers (Malvern Mastersizer2000, Malvern Instruments Ltd., Malvern, UK).

## 4. Results and Discussion

### 4.1. Model Simulation under Different Fluxes in the Lab-Scale A^2^/O-MBR

**Figure 4:**
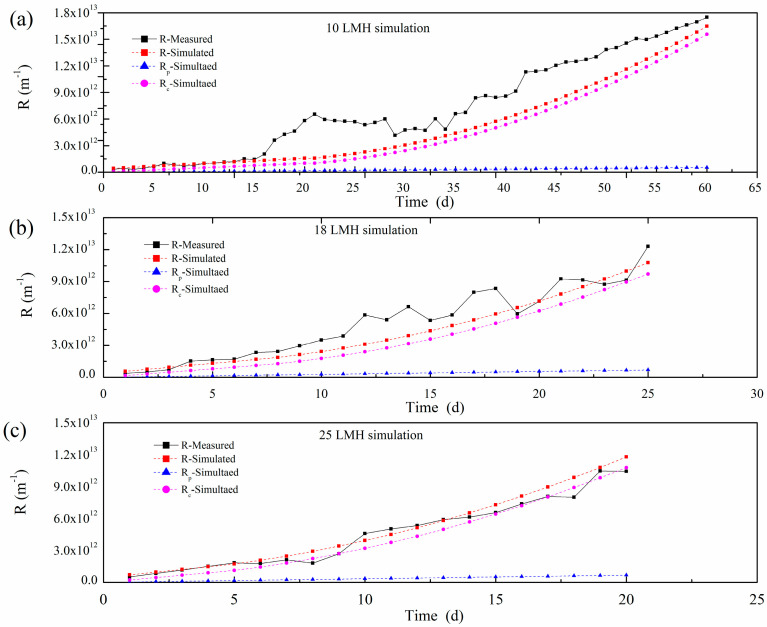
R simulations under different fluxes. (**a**) 10 LMH; (**b**) 18 LMH; (**c**) 25 LMH s.

TMP and flux were monitored along time under different filtration fluxes, and the polymer film resistance R was calculated by Darcy law. With consideration of the sludge’s attachment to and detachment from the polymer film surface, the long-term resistance model developed in this study is capable of simulating polymer film fouling of the lab-scale A^2^/O-MBR. The individual fouling components and total polymer film resistance were obtained by simulation, and the resistances under different fluxes are shown in Figure 4.

From Figure 4, we can note that the simulation results are comparable with the experimental observations on the trend of polymer film development under different operation fluxes. For the individual component of polymer film fouling resistances, the resistance caused by sludge cake accumulation (R_c_) was the dominant component of the total filtration resistance R. It is noted that R_c_/R was less than 30% at the initial stage, while it was over 90% at the end of the simulation, which indicates that sludge cake accumulation and compression were sustained. Pore fouling resistance R_p_ was also increased along with the simulation or experiment time, although the contribution of R_p_ to R varied from 10% at the initial stage to 3% at the end of the simulation.

The comparison of the simulated and experimentally measured resistances under different operations is shown in Table 3. At the end of the operation time, the errors of the fouling resistance R between the simulated and experimental measurements were less than 13.5% under the different fluxes, which implied the availability and the good accuracy of the long-term membrane resistance model when used in the simulation of the fouling resistances in MBR operation. The simulated values of pore fouling resistance R_p_ were lower than the measured values, which resulted from the constant SMP concentration and lower value of θ set in the model, while, in fact, the SMP concentration could not be an exact constant during the A^2^/O-MBR’s operation.

According to the long-term membrane resistance model, the polymer film resistances are directly influenced by t flux, TMP, sludge and SMP concentrations, and some operational are parameters listed in Table 2. A faster resistance growth could have resulted from higher flux. It was noted that the experiments with higher flux usually ended at a lower total resistance R, along with a higher pore fouling resistance R_p_. Conversely, under a lower flux, the experiments always finished at a higher cake layer resistance R_c_ accompanied with a lower pore fouling resistance R_p_. It means that the flux affected the pore fouling, and more foulants entered into the polymer film pore under the suction drag force. The cake layer resistance was influenced by the flux, sludge concentration and aeration intensity. In this study, we found that flux is an important factor for cake layer resistance. Though R_c_ was higher under the operation of a lower flux and lower TMP, the reactor could still run for a long time, which may be related to the difference in the bio-cake structure caused by cake accumulation and compression [19].

### 4.2. The Determination of Sustainable Critical Flux

The main objective of this paper was to predict the sustainable critical flux of MBR by using the long-term membrane resistance model. To search for the sustainable critical flux of MBR, the total polymer film resistance, R, with operation time were simulated by using the Equations (16) and (17). Under the conditions of different fluxes from the subcritical flux (10 LMH) to the supercritical flux (35 LMH), temperatures were set at 15 and 25 °C, and the sludge concentration was set at 8000 mg/L, with other parameters as shown in Table 2.

The specific flux actually decreased with the resistance growth and increase in TMP; thus, it can be used to measure the degree of polymer film fouling. One study has suggested that the polymer film should be cleaned while the specific flux is below 0.5 LMH/kPa [40]. According to the Darcy law, the relationship between polymer film resistance and the specific flux can be expressed as Equation (18)

(18) $$J_{s}=u\frac{1}{\mu }\cdot \frac{1}{R}$$

where $J_{s}$ is the specific flux, LMH/kPa. R is the total polymer film resistance, m^−1^. u is the unit conversion factor.

**Figure 5:**
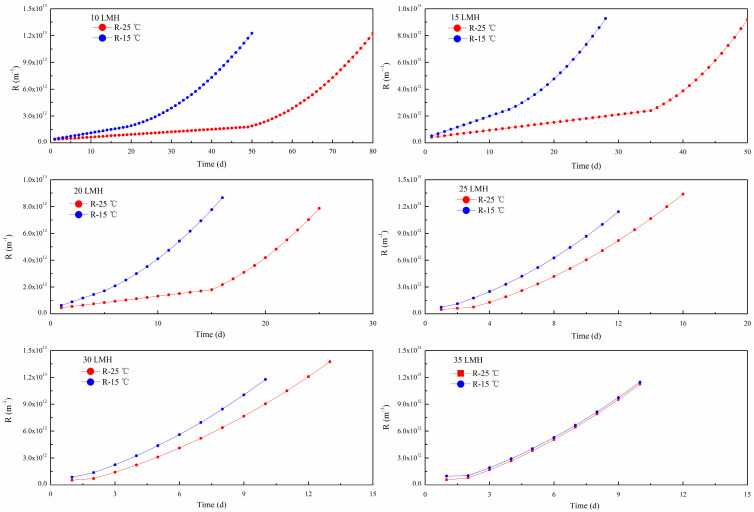
Total polymer film resistances under different fluxes and different temperatures.

**Figure 6:**
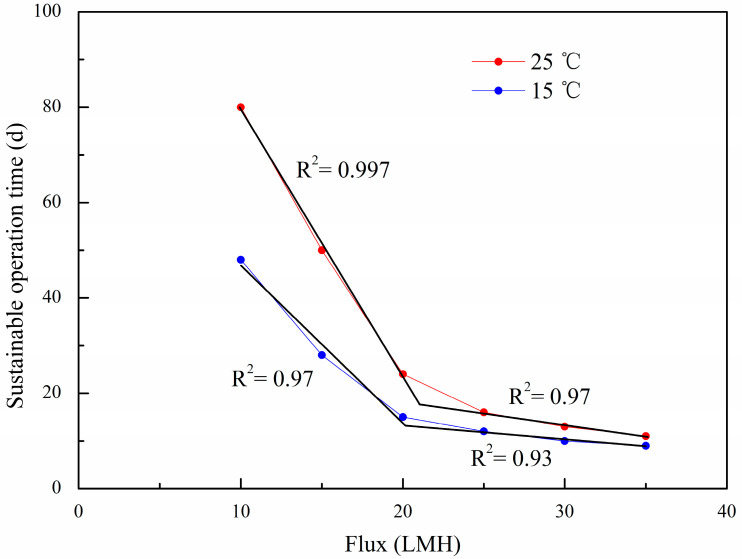
Relationship between flux and sustainable operation time.

According to the above suggestion, each simulation of MBR in this study was stopped as the specific flux reached 0.5 LMH/kPa, and the actual operation time was taken as the sustainable operation time for the given operation conditions. The total resistance curves of the modelling results are given in Figure 5, and the relationship between the flux and sustainable operation time is shown in Figure 6.

The total resistance under different fluxes was simulated by the long-term membrane resistance model. Simulated conditions were varied with different operational fluxes from the subcritical flux to the supercritical flux, 10 to 35 LMH, respectively, a sludge concentration of 8000 mg/L, and other simulated parameters as shown in Table 1. Then the sustainable operation time was predicted and is shown in Figure 6.

From Figure 5 and Figure 6, it can be seen that when the fluxes are in the range of 10~20 LMH, the differences in the sustainable operation times at the temperatures between 25 and 15 °C are significant. However, with the increase in flux, the sustainable operation times become shorter and their differences become smaller, and in particular, as the fluxes rise up to 35 LMH, the sustainable operation times reduce to about 10 days without a great difference at different temperatures. The result shows that temperature had an obvious effect on the total polymer film resistance at a lower flux than that at a higher flux, and the sustainable operation time under 25 °C was twice that under 15 °C. However, the differences between 25 and 15 °C were gradually narrowed with an increase in the flux. This indicates that the operation’s flux may have more influence on polymer film fouling than the temperature.

The relationship between the flux and the sustainable operation time was analyzed based on the modelled data above (Figure 6). Some studies showed that the relationship between the flux and operation period was a linear correlation under subcritical fluxes in a flat-sheet MBR [24], or in a hollow fiber MBR [41]. However, different to the result of the linear correlation suggested by Wang and Guglielmi, it was approximately regarded as an exponential correlation in this study. The equations obtained under 25 and 15 °C can be expressed as Equations (19) and (20) with correlation coefficients R^2^ 0.985 and 0.993, respectively.

(19) $$T_{25\;℃}=265.85\times e^{\left(-\frac{J}{7.86}\right)}+6.51$$

(20) $$T_{15\;℃}=185.94\times e^{\left(-\frac{J}{6.56}\right)}+7.83$$

where $T_{15\;℃}\;and\;T_{25\;℃}$ are the sustainable operation times at the temperatures of 25 °C and 15 °C, respectively, d, and J is the polymer film flux, LMH.

In Figure 6, the exponential curve can be divided into two parts, which can be fitted for two straight lines, respectively. The impact of flux on the sustainable operation time could be associated with the slope of the straight line. It is interesting to note that there is an intersection of two straight lines. When the operation flux is above this intersection value, the sustainable operation time will be shortened dramatically, and if the operation flux is below this value, the sustainable operation time will be increased significantly. The intersection of the two straight lines could be regarded as the sustainable critical flux value. Compared to the critical flux, the sustainable critical flux reflected the real relationship between the flux and the operation time during a long-term run, and narrowed the range of the subcritical flux.

The sustainable critical flux can be obtained as 20 LMH by the method described above. A frequent polymer film cleaning procedure must be conducted if the operation flux is higher than 20 LMH. It is interesting that the sustainable critical flux can be taken as the same value of 20 LMH at 25 and 15 °C, but the difference is the sustainable operation time is 25 d at 25 °C, while it is only 15 d at 15 °C. That implies that when the sustainable critical flux is determined, the temperature will affect the sustainable operation time, which is shorter at a lower temperature and longer at a higher temperature.

It is also worth noting that the sustainable critical flux obtained is at 67% of the critical flux detected in this study, and it is comparable with the “proper flux” at 56% of the critical flux obtained in the study of Wang et al. [24]. The result indicates that the critical flux was too large to be used as the sustainable operation flux. Compared with the well-known definition of critical flux, the sustainable critical flux is much more available and more practical for the design and operation of an MBR than the critical flux or subcritical flux. This is because the sustainable critical flux not only reflects the real relationship between the flux and operation time during the long-term operation of an MBR very well, but also the temperature’s influence on it.

### 4.3. Model Application in a Large-Scale A^2^/O-MBR Municipal Wastewater Treatment Plant

The model’s simulation and application was conducted in a large-scale A^2^/O-MBR WWTP in Beijing. The scale of the WWTP is 15,000 m^3^/d, and hollow fiber polymer film modules are used with a total polymer film area 396,000 m^2^. The polymer film operation parameters include alternate aeration with low and high intensities, and chemically enhanced backflush (CEB). The average intensity of high aeration is about 180 Nm^3^/m^2^h, while the low aeration intensity is about 80 Nm^3^/m^2^h. The ratio of alternate aeration time between low and high intensity is 4:1. CEB frequency is once a week with a NaClO concentration of 1500 mg/L. The average operation flux is 20 LMH. R_m_ of the hollow fiber polymer film is 6 × 10^11^. The average concentration of SMP is about 20 mg/L. According to the operation’s condition, the parameters of the polymer film resistance model were calculated and are shown in Table 4.

CEB has positive effects on polymer film fouling control [42]. However, the mechanism of CEB is not clear. To investigate this, the effect of periodic CEB was assumed as uniform for polymer film resistance control. Two parameters, b1 and b2, were introduced into the model to represent the percent of $R_{p}$ and $R_{c}$ residue after CEB, respectively. Thus, the Equations (4), (16) and (17) can be expressed as Equations (21)–(23), respectively.

(21) $$R_{p}=r_{SMP}JC_{SMP}t\theta b1\;$$

(22) $$R_{c}=r_{c}\left(Et_{1}-Kt_{2}\right)\mathrm{CJ}\varepsilon \tau$$

(23) $$R_{comp}=r_{comp}\left(Et_{1}-Kt_{2}\right)\mathrm{CJ}\varepsilon \tau b2$$

The data of three polymer film modules were used to determine b1 and b2, and b1 = 0.05, b2 = 0.95 were chosen as the proper parameters for simulation (the details of b1 and b2 determination are shown in the Supplementary Materials). The b1 value means that the CEB has a significant effect on polymer film pore clogging control, and almost 95% of $R_{p}$ can be removed by CEB, while for b2, the CEB has little effect on cake layer control, as only 5% of $R_{c}$ can be removed.

**Figure 7:**
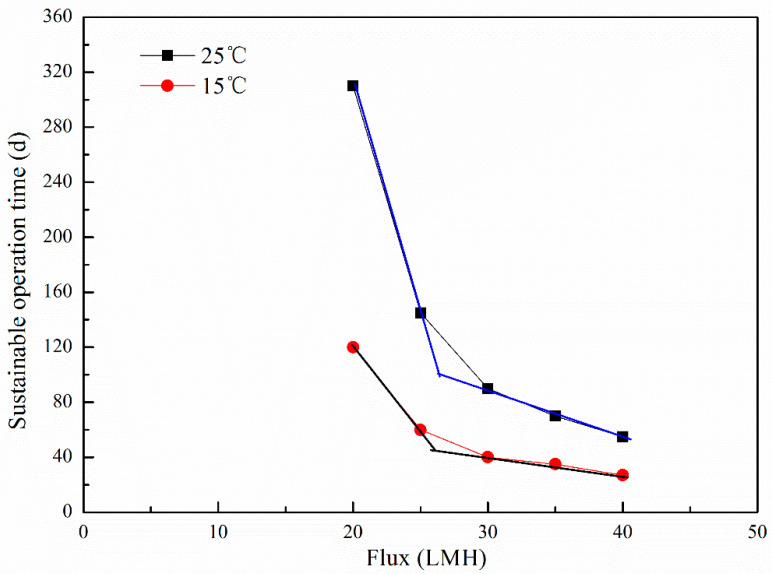
Relationship between flux and sustainable operation time of WWTP.

The total resistances under different fluxes of the WWTP were simulated by the long-term membrane resistance model, and the relationship between the flux and the sustainable operation time was analyzed based on the simulation data as in the methods above. The simulated conditions varied the operational flux from a subcritical flux to a supercritical flux, 20 to 40 LMH, respectively, with a sludge concentration of 6000 mg/L, and other simulated parameters as shown in Table 3. Then the sustainable operation time was predicted and is shown in Figure 7.

Calculating the sustainable critical flux using the data from Figure 7, it can be found that the sustainable critical flux was 26 LMH, and the polymer film module can operate stably for more than 110 days at 25 °C, while it can only operate for 48 days at 15 °C. To achieve the same operation time as that at 25 °C, the sustainable critical flux was 20 LMH at 15 °C, and this is only 75% of the sustainable critical flux at 25 °C. As we know, two factors influence the polymer film area, the operation flux and the capacity of the WWTP. In northern China, winter is the dry season with a low temperature, and should be especially considered for the determination of the polymer film area.

## 5. Conclusions

A long-term membrane resistance model was developed to simulate polymer film fouling of an MBR successfully. Drag force by suction, lifting force by aeration and adhesive force by stickiness were considered in the model development. The total polymer film fouling resistance in the model was decomposed into the individual components of pore fouling resistance, sludge cake accumulation and cake layer compression resistance. The model can effectively simulate the fouling phenomenon in an MBR at different fluxes. Considering the influence of temperature, the model was calibrated by temperature coefficient τ, and a good result was obtained. Additionally, the effect of flux on the polymer film operation time can be determined, and the results indicated that there was an exponential correlation between flux and operation time. The exponential curve was divided into two parts, and fitted to two straight lines, respectively, and the intersection of the two straight lines was regarded as the sustainable critical flux. The sustainable critical flux obtained in this study was just 67% of the critical flux in this study. Compared with selected operation fluxes by critical flux testing, the sustainable critical flux provides more information that is practical for the design of MBRs. In addition, the proposed model was used to simulate a full-scale WWTP of A^2^/O-MBR, and the sustainable critical flux was 26 LMH.

## Figures and Tables

**Table 1:** Comparison of resistances before and after temperature calibration.

Measured Value (m^−1^)	Simulated Value (m^−1^)		Standard Deviation (%)	
	Before Calibration	After Calibration	Before Calibration	After Calibratration
1.18 × 10^12^	2.73 × 10^12^	1.32 × 10^12^	101.57 ± 63.90	7.18 ± 21.86

**Table 2:** Parameters and coefficients used in the modelling and simulations.

Symbol	Meaning	Value	Source of Data
C	Sludge concentration, (MLVSS, g/L)	6.5	Measured
C_SMP_	SMP concentration, (TOC, mg/L)	37.7	Measured
g	Gravitational constant (m/s^2^)	9.81	Constant
J	Flux (m^3^/(m^2^•d))	——	Operation parameter
K_1_	Coefficient (m)	4 × 10^−6^	Constant (from literature)
q	Aeration intensity (L/(m^2^•s))	0.83	Operation parameter
R_m_	Polymer film intrinsic resistance (m^−1^)	3.74 × 10^11^	Measured
r_SMP_	Specific pore fouling resistance (m/kg)	9.1 × 10^11^	Measured
r_c_	Specific filtration resistance of cake laye (m/kg)	3.14 × 10^11^	Measured
TMP_c_	Critical pressure (kPa)	10	Measured
t_1_	Filtration time (d)	6 min filtration and 1 min stop cycle, t_1_ = 6/7t_2_.	Operation parameter
t_2_	Aeration time (d)		Operation parameter
α	Stickiness of biomass particles or stickiness between biomass particles and polymer film surface	At sludge accumulation stage, α = 0.1; at cake layer compression stage, α = 0.5	Constant (from literature)
γ	The ratio of boundary velocity to flow velocity	0.02	Constant (from previous work)
ε	The percent of sludge practice size less than 100 μm	0.6	Measured
μ	Viscosity of the sludge suspension (mPa•s)	5.5	Measured
θ	Coefficient of polymer film pore clogging caused by SMP	1.25 at 10 LMH, 1.5 at 18 and 25 LMH	Constant (from previous work)
ρ	Density of the sludge suspension (kg/m^3^)	1.0 × 10^3^	Constant

**Table 3:** Comparison of resistances between experimental measurements and simulation under different operations.

Operational Flux	Measured Value (m^−1^)			Simulated Value (m^−1^)			Error (%)		
	R_p_	R_c_	R	R_p_	R_c_	R	R_p_	R_c_	R
10 LMH	5.9 × 10^11^	1.58 × 10^13^	1.75 × 10^13^	5.30 × 10^11^	1.55 × 10^13^	1.65 × 10^13^	−10.2	−1.9	−5.7
18 LMH	8.7 × 10^11^	1.01 × 10^13^	1.13 × 10^13^	6.84 × 10^11^	9.72 × 10^12^	1.07 × 10^13^	−21.4	−3.8	−5.3
25 LMH	7.3 × 10^11^	9.26 × 10^12^	1.04 × 10^13^	6.78 × 10^11^	1.08 × 10^13^	1.18 × 10^13^	−7.1	16.6	13.5

**Table 4:** Parameters of polymer film resistance model.

Parameters	r_c_ (m/kg)	r_p_ (m/kg)	γ	E	K	TMP_c_ (kPa)
High aeration intensity	2.97 × 10^11^	8.30 × 10^11^	0.01	0.125	0.059	14~15
Low aeration intensity				0.087	0.086	

## Data Availability

Not applicable.

## Supplementary Materials

The following supporting information can be downloaded at: https://www.mdpi.com/article/10.3390/polym15102319/s1, Figure S1: R simulation regardless of CEB; Figure S2: R of simulation and measurement under different coefficients; Figure S3: R simulation and measurement of membrane module B and C. Table S1:Coefficients of different simulation process.
