# Supplementary material for: Determination of Sustainable Critical Flux through a Long-Term Membrane Resistance Model
Source: Polymers (Basel). 2023 May 16;15(10):2319. doi: 10.3390/polym15102319 (PMC10220670; doi:10.3390/polym15102319)
Supplement: Supplementary file 1 [file polymers-15-02319-s001.zip › polymers-2327278-supplementary.pdf]

polymers

MDPI

Supplementary data

Determination of Sustainable Critical Flux through a Long-Term Membrane Resistance Model

Rongle Xu [1,2,*], Yaobo Fan [3], Min Yang [3] and Jinqiu Song [2]

[1] Scientific Research Academy of Guangxi Environmental Protection, Nanning 530022, China

[2] School of Resources, Environment and Materials, Guangxi University, Nanning 530004, China; xurongle@gxhky.org (R.X) and 17745056451@163.com (J.S.)

[3] Research Center for Eco-Environmental Sciences, Chinese Academy of Sciences, Beijing 100085, China; ybfan@rcees.ac.cn (Y.F.); yangmsunb@126.com (M.Y.)

* Correspondence: xurongle@gxhky.org

Model application in a large-scale A2/O-MBR municipal wastewater treatment plant b1 and b2 determination

(1) R simulation regardless of CEB

Choosing an arbitrary membrane module A for R simulation, regardless of CEB, the simulation and measurement value was shown in Fig. S1.

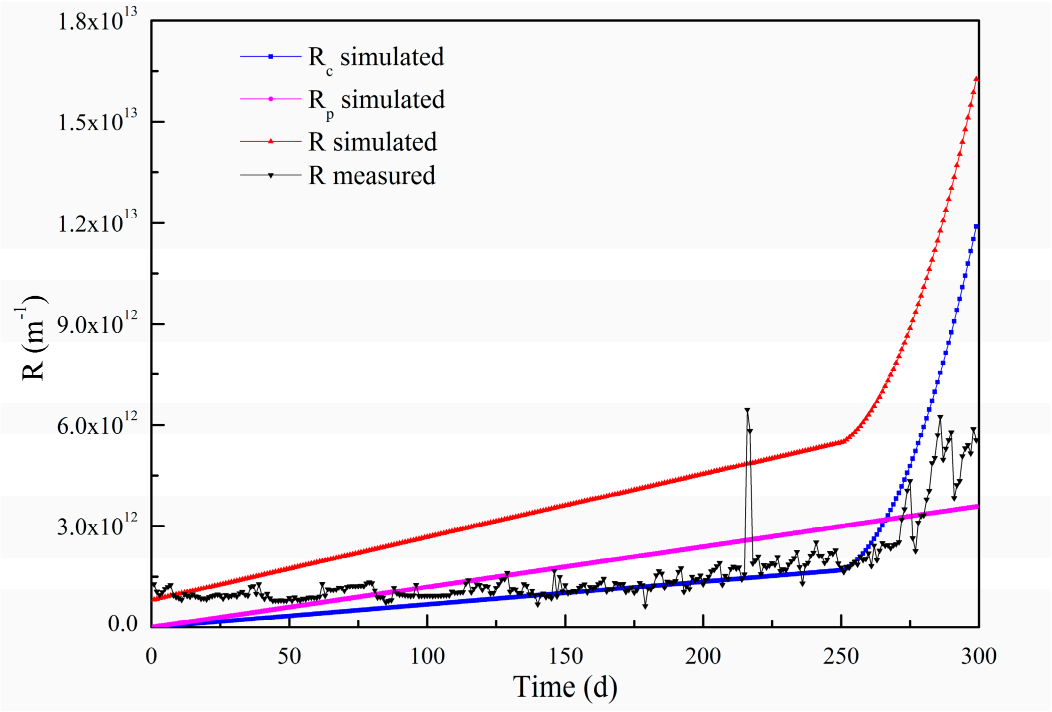

Figure S1. R simulation regardless of CEB.

From the Fig. S1, it was found that R-simulated was well above the R-measured. However, the R_c-simulated was consistent with R-measured, which means Rp-simulated was overestimated, and CEB had a positive effect on pore clogging remove. Therefore we considered b1, the parameter of Rp residue after CEB, was greater than b2, the parameter of Rc residue after CEB.

(2) Determination of b1 and b2

According to the stronger inhibition of membrane pore resistance by CEB, different coefficients of b1 and b2 were set as Table 1s and simulated. The simulated results were shown in Fig, S2.

Table S1. Coefficients of different simulation process.

Coefficients	1	2	3	4	5	6
b1	0.05	0.05	0.1	0.1	0.15	0.2
b2	0.95	0.9	0.9	0.8	0.8	0.7

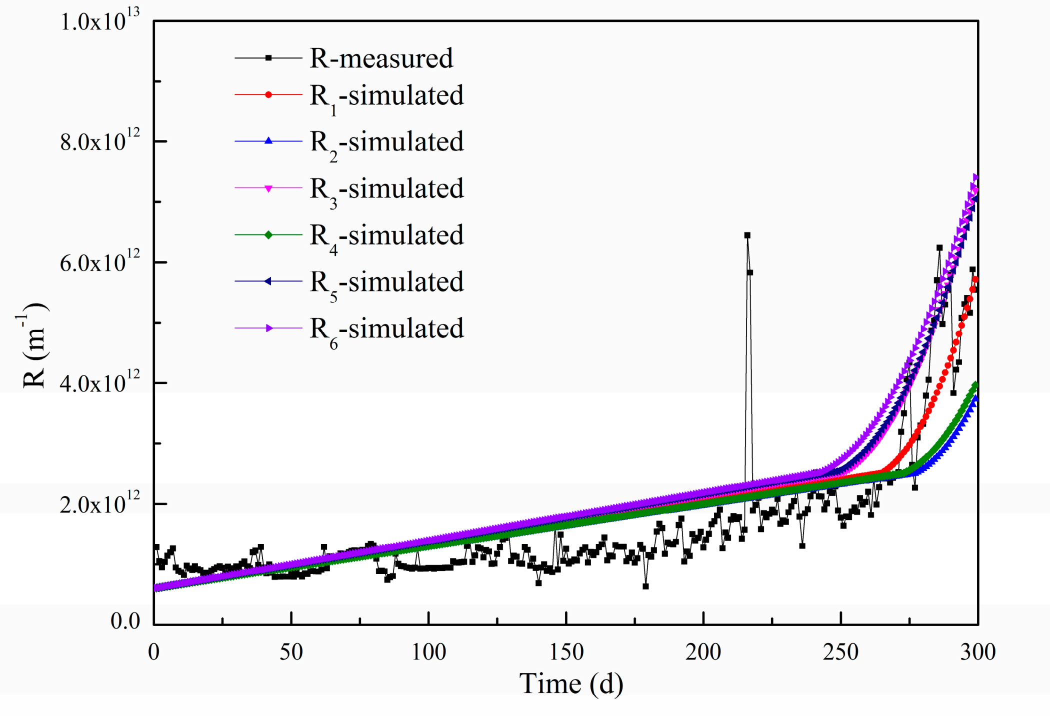

Figure S2. S R of simulation and measurement under different coefficients.

It can be found that R_1-simulated was very consistent with R-measured from the Fig. S2. The simulation of R3, R5 and R6 were higher than R-measured, and that of R2, R4 were lower. b1=0.05, b2=0.95 was more suitable for simulation.

(3) Calibration of b1 and b2

In order to verify the reliability of b1 and b2, R of two other membrane modules B and C were simulated, and the results was shown in Fig. 3S.

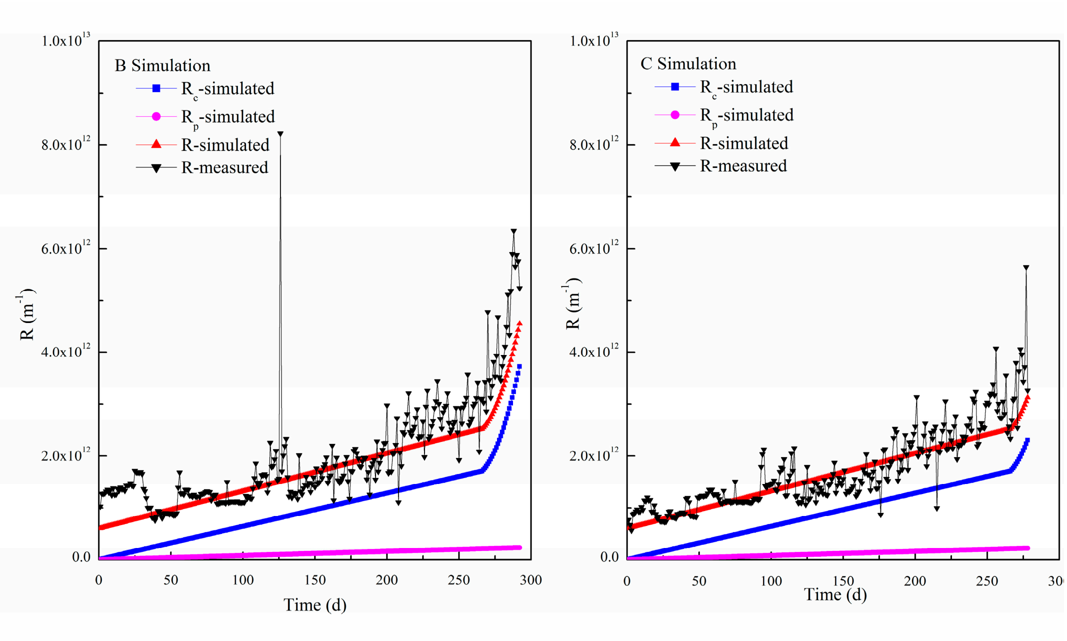

Figure S3. S R simulation and measurement of membrane module B and C.

The R-simulated of B and C were very consistent with R-measured, which means that b1=0.05, b2=0.95 were proper parameters for R simulation with CEB.
